# P4VP Modified Zwitterionic Polymer for the Preparation of Antifouling Functionalized Surfaces

**DOI:** 10.3390/nano9050706

**Published:** 2019-05-07

**Authors:** Chaoqun Wu, Yudan Zhou, Haitao Wang, Jianhua Hu

**Affiliations:** State Key Laboratory of Molecular Engineering of Polymers, Department of Macromolecular Science, Fudan University, 2005 Songhu Road, Shanghai 200438, China; cqwu16@fudan.edu.cn (C.W.); amandazhou2012@163.com (Y.Z.); wanght@fudan.edu.cn (H.W.)

**Keywords:** copolymer, biofilms, spin-casting, antifouling

## Abstract

Zwitterionic polymers are suitable for replacing poly(ethylene glycol) (PEG) polymers because of their better antifouling properties, but zwitterionic polymers have poor mechanical properties, strong water absorption, and their homopolymers should not be used directly. To solve these problems, a reversible-addition fragmentation chain transfer (RAFT) polymerization process was used to prepare copolymers comprised of zwitterionic side chains that were attached to an ITO glass substrate using spin-casting. The presence of 4-vinylpyridine (4VP) and zwitterion chains on these polymer-coated ITO surfaces was confirmed using 1H NMR, FTIR, and GPC analyses, with successful surface functionalization confirmed using water contact angle, X-ray photoelectron spectroscopy (XPS), and atomic force microscopy (AFM) studies. Changes in water contact angles and C/O ratios (XPS) analysis demonstrated that the functionalization of these polymers with β-propiolactone resulted in hydrophilic mixed 4VP/zwitterionic polymers. Protein adsorption and cell attachment assays were used to optimize the ratio of the zwitterionic component to maximize the antifouling properties of the polymer brush surface. This work demonstrated that the antifouling surface coatings could be readily prepared using a “P4VP-modified” method, that is, the functionality of P4VP to modify the prepared zwitterionic polymer. We believe these materials are likely to be useful for the preparation of biomaterials for biosensing and diagnostic applications.

## 1. Introduction

Biological pollution [1,2,3,4], which is affected by the adsorption and aggregation of proteins, microorganisms, and bacteria on the surface of materials, is a problem that is often encountered in the field of medical diagnostics, medical transplantation, and dock materials. For example, the biological molecules that adhere to biomaterial surfaces and the biofilm formation on material surfaces are two major causes of implanting operation failure. Therefore, the development of highly efficient nonfouling materials, such as those resistant to protein and microorganisms/bacteria, is always a focus of research [5,6,7,8,9].

In recent years, studies have found that the repeating units of zwitterionic polymers contain a positive group and a negative group, which have excellent resistance to protein and microbial/bacterial adsorption [10]. Polysulfobetaine (PSB), polycarboxybetaine (PCB), and their copolymers are zwitterionic polymers that contain similar numbers of negative and positive groups [11,12,13]. The repulsive forces between the polymers induce the formation of highly hydrated layers, which have been shown to be responsible for the nonfouling properties of these polymers [14,15]. These hydrated layers are formed by the electrostatic attractions within these amphoteric polymers, which are stronger and more stable than the simple hydrated layers present in the poly(ethylene glycol) (PEG) polymers that are formed by simple hydrogen bonding forces [16,17,18]. Therefore, zwitterionic polymers generally exhibit better nonfouling performance than PEG polymers [14], with zwitterionic polymers shown to exhibit outstanding resistance against protein and cell adsorption in numerous biological systems. Even under complicated conditions such in serum or blood, zwitterionic polymers can exhibit outstanding resistance against protein and microorganisms/bacteria adsorption [15]. In addition, zwitterionic polymers have thermal stability, chemical stability, biocompatibility, and blood compatibility properties [19,20]. Therefore, zwitterionic polymers are expected to become the new generation of nonfouling biomaterials.

Although the antifouling performance of zwitterionic polymers where their resistantance to nonspecific protein adhesion, microbial/bacterial adsorption, and biofilm formation, can allow them to effectively avoid bacteria adhering on their surfaces, due to their high water solubility and certain hydrolysis properties, the film formation of these kinds of polymers is poor, which greatly limits the application of zwitterionic membranes in antifouling films [21,22]. Zwitterionic polymers exhibit some disadvantages in poor mechanical properties and strong water absorption, which may result in the inability to use their homopolymer directly for films. Thus, in recent years, some researchers have focused their research on the surface grafting technique used for their preparation and the generation of the polymers’ architecture. In previous work, our group used spin-casting processes to attach asymmetric amphiphilic PEG/polystyrene(PS) polymer brush coatings to solid surfaces. These PEG/PS polymers exhibited good nonfouling properties; however further optimization is required to broaden the range of antifouling properties of these polymers. In this work, we introduced the bio-inert poly(4-vinylpyridine) (P4VP) to modify zwitterionic polymers for the preparation of biofilms. The pendant group of P4VP is a six-membered aromatic heterocyclic ring. The N atom has a pair of unbonded electrons in the SP_2_ orbital plane [23]. Its water solubility is poor, but the film has good toughness and high strength. The reaction can easily produce zwitterionic polymers [24,25,26]. Therefore, this paper uses the functionality of P4VP to modify the prepared zwitterionic polymers, and optimize the biofouling performance of the film by regulating the degree of polymerization and modification ratio.

## 2. Materials and Methods 

### 2.1. Materials

2-(N,N′-dimethylamino)ethyl methacrylate (DMAEM, 98%), 4-vinylpyridine (4VP), 2,2’-Azobis(2-methylpropionitrile) (AIBN), 2-(Dodecylthiocarbonothioylthio)-2-methylpropionic acid, 98%, 1-aminopropane (98%), Bovine serum albumin (BSA, 99%), and lysozyme (Lys, 90%) were purchased from Aladdin (Aladdin Industrial Corporation, China, Shanghai) and the inhibitors were removed by passing the samples through a plug of activated basic aluminum oxide. AIBN was recrystallized twice from ethanol. N,N-Dimethylformamide (DMF, 99.8%) SuperDry was provided by J&K Scientific Ltd (China, Beijing). ITO glasses were purchased from Zhuhai Kaiwei Photoelectric Co., Ltd., 100 mm × 100 mm × 1.1 mm, <10 ohm/sq. Surface Plasmon Resonance (SPR) Au chips were purchased from Biosensing Instruments. Dulbecco’s modified Eagle medium (DMEM/HIGH, GLUCOSE 1X) was purchased from Hyclone, GE Life Science. Fetal bovine serum (FBS) was purchased from Gibco, Thermo Fisher Scientific. Cell culture reagents were purchased from Invitrogen (China, Shanghai). Acetone was dried by distillation from anhydrous calcium sulfate (CaSO4). All other chemicals were used as received unless otherwise stated.

### 2.2. Synthesis of the P4VPPC-co-PDMAPC Copolymer 

**Scheme 1:**
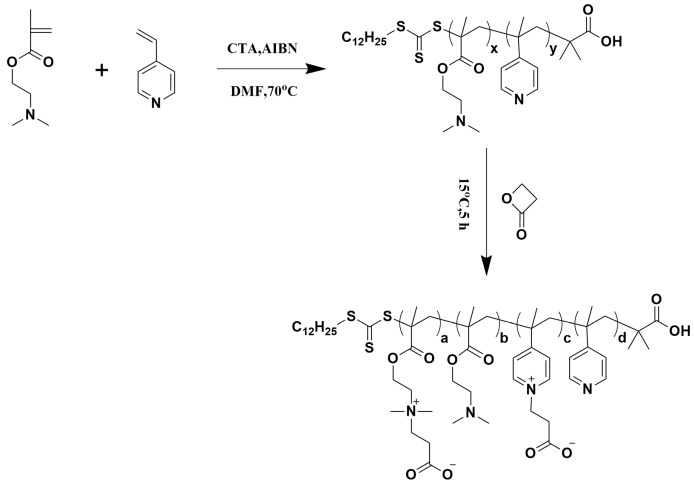
Synthesis of the antifouling P4VPPC-co-PDMAPC copolymer.

Scheme 1 is the synthesis of the antifouling P4VPPC-co-PDMAPC copolymer. The 4VP and DMAEMA random copolymers were named as P4VP-co-PDMAEMA. After P4VP-co-PDMAEMA reacted with β-Propiolactone, the copolymers were named as P4VPPC-co-PDMAPC. The P4VPPC-co-PDMAPC copolymer was synthesized by reacting the P4VP-co-PDMAEMA random copolymers, that were prepared according to the method published elsewhere, with β-propiolactone as follows [27]. A certain amount of β-Propiolactone in dry acetone was added dropwise to a solution of P4VP-co-PDMAEMA dissolved in dry acetone, and the mixture was stirred under a nitrogen atmosphere at 15 °C for 5 h. The obtained product was rinsed with anhydrous acetone, dissolved and dialyzed with methanol, and then dried under vacuum conditions. In order to investigate the effect of the P4VP-co-PDMAEMA molecular weight and β-propiolactone content on the film-forming and antifouling properties, we prepared polymers with different molecular weights and different modification ratios. P1, P2, P3 and P4 represent the different molecular weights of the P4VP-co-PDMAEMA polymers, respectively; CP20, CP40, and CP60 represent the P4VPPC-co-PDMAPC copolymers with a degree of zwitterionic modification of 20%, 40%, and 60%, respectively.

### 2.3. Synthesis of the Polymer Films

The commercially available ITO glass was cut into a uniform size of 25 mm × 25 mm, soaked in a deionized water, acetone, and ethanol solution for 30 min, rinsed with ethanol, and dried under vacuum overnight. A 20 mg/mL solution of the P4VP-co-PDMAEMA and P4VPPC-co-PDMAPC polymers in ethanol was prepared and the solution was passed through a 0.45 μm filter after thorough stirring. Before spin-coating, the ITO surfaces were purged with nitrogen gas to remove surface contaminants. The adjustment speed of the film formation rate was set at 2500 rpm for 30 s. The amount of surface solution was controlled as consistently as possible. Each copolymer sample was repeatedly spin-coated on different ITO glasses three times for surface characterization to ensure that the test was effective. The polymer films were dried overnight in vacuum drying [28,29]. Similarly, the pure gold chip used in the SPR test was prepared after the pretreatment, using the same polymer concentration and spin-coating parameters.

### 2.4. Polymer Characterization

All 1H NMR spectra were recorded on a JEOL resonance ECZ 400S spectrometer (400 MHz, JEOL Ltd., Tokyo, Japan) and analyzed with MestReNova LITE software. FTIR spectra were recorded on a Nicolet AVATAR 360 spectrophotometer. The liquid sample was directly tested by the liquid membrane method; the solid sample was subjected to the KBr tablet method. All infrared spectra are accumulated 32 times with a 4 cm-1 resolution. Relative molecular weight and molecular weight distribution were measured by a conventional gel permeation chromatography (GPC) system equipped with a Waters 1515 Isocratic HPLC pump, a Waters 2414 refractive index detector, and a set of Waters Styragel columns (HR3 (500–30,000), HR4 (5,000–600,000), and HR5 (50,000–4,000,000), 7.8 × 300 mm, particle size: 5 µm). GPC measurements were carried out at 35 °C using THF as eluent with a flow rate of 1.0 mL/min. The system was calibrated with linear polystyrene standards [30,31,32].

### 2.5. Surface Characterization

Water contact angles were measured at ambient temperature using a CA system (OCA20, Dataphysics, Germany) to characterize surface hydrophilic properties. 2 μL water droplets were then added to the surface using a microliter syringe. Contact angles were determined as the average value of three different measurements determined at different locations on the surface. XPS analysis was carried out using a PHI5300 (PerkinElmer) spectrometer using Mg Kα excitation radiation (hν = 1253.6 eV), with the binding energy scale referenced to the C1s peak at 284.6 eV. Surface morphology of the formed polymer layers were observed using tapping mode AFM (Multimode 8, Bruker), with 3D images with a scan size of 2 μm analyzed using Nanoscope software.

### 2.6. Protein Adsorption Test

SPR sensors were used to measure the protein adsorption on polymer surfaces using a BI-2000 (Biosensing Inc., USA.) Kretschmann SPR system. Bare gold films were soaked in an anhydrous acetone solution overnight prior to use to prevent possible contamination of the surface of the gold film. After repeated washing with ultra-pure water, the surfaces of the chips were dried with nitrogen, before further structural modification was carried out. In these experiments, a sensing chip was placed above the prism of a SPR system, with optical matching oil used to ensure refractive index matching of the two components. 0.15 mL of a PBS solution (pH = 7.4) was flowed over the chip surface to obtain a stable baseline, after which 1.0 mg/mL solutions of BSA and lysozyme in PBS (0.15 M, pH 7.4) were added at a flow rate of 0.05 mL/min. After the SPR response of the deposited protein substrates had reached a stable value, the surface was washed with PBS solution and the changes in the SPR response were determined. The wavelength shifts between the buffer baselines were established before and after protein injection to determine the changes in the surface protein concentration. The amount of protein adsorbed on the surfaces was quantified through the resonance angle shift (△θ, mDeg), with a bare Au substrate used as the control. Each sample of the protein adsorption was tested for three repetitions.

### 2.7. Cell Adhesion Test

The antifouling interactions between the polymer brush coatings and the pollutants were studied using cell adhesion experiments to determine the interactions with the human embryonic kidney cell line (HEK-293T) cells. HEK-293T cells were supplied by the Chinese Science Academy (Shanghai, China) and grown using high-glucose DMEM supplemented with 10% (v/v) FBS and 1% antibiotics (penicillin/streptomycin, 100 U/mL) at 37 °C, under a 5% CO_2_ atmosphere. All culture equipment was sterilized in an autoclave before use. Polymer brush films were sterilized by treatment with UV light overnight and then placed in 35 mm glass-bottomed dishes. HEK-293T cells (1.0 × 10^4^ cell/well) were seeded into each well and incubated at 37 °C for 6 h. Unattached cells were removed by washing three times with PBS solution, and imaged using microscopy. Each sample of the cell test was tested for three repetitions.

## 3. Results

### 3.1. Characterization of the P4VPPC-co-PDMAPC Copolymer

**Figure 1:**
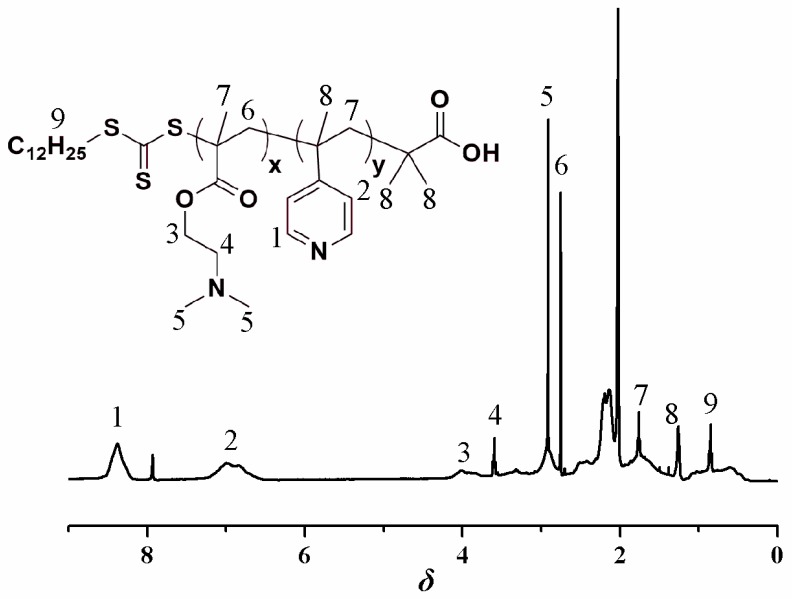
1H NMR spectrum of P4VP-co-PDMAEMA (P4) in Acetone-d_6_.

**Figure 2:**
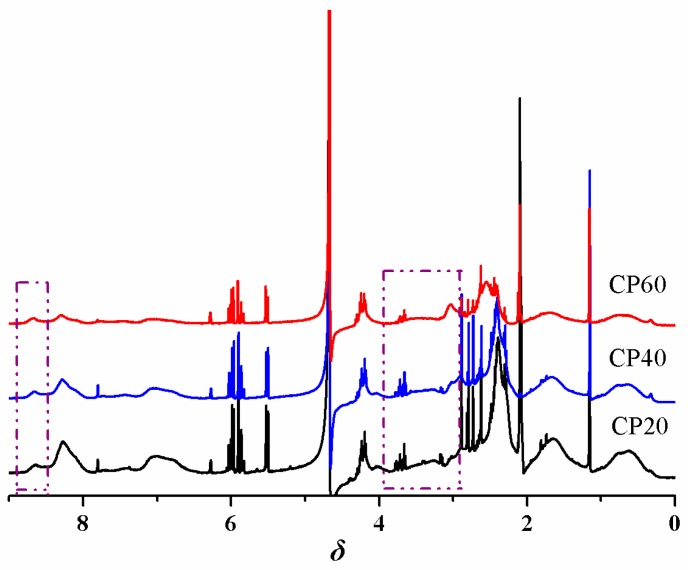
1H NMR spectrum of CP20, CP40, and CP60 in D_2_O.

**Figure 3:**
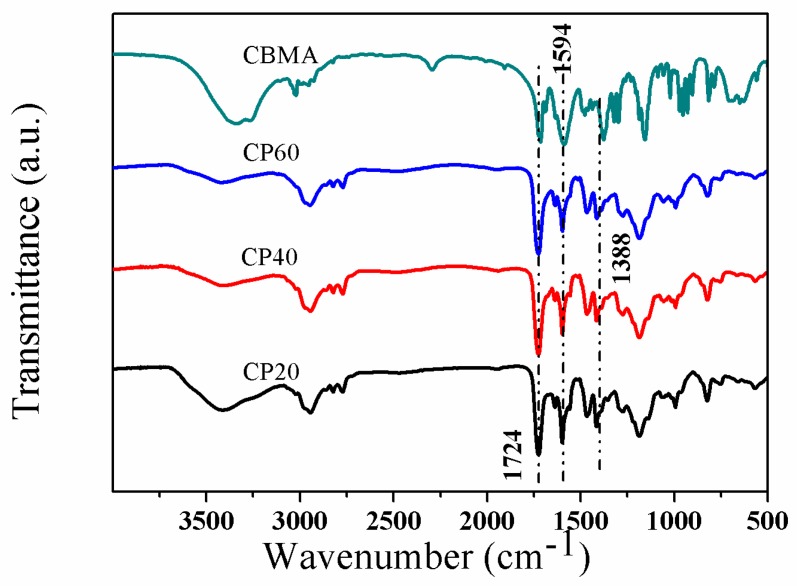
FTIR spectra of CBMA, CP20, CP40, and CP60.

The P4VPPC-co-PDMAPCs were prepared using a two-step process involving RAFT copolymerization and subsequent derivatization with β-propiolactone. In the first step, 2-(N,N′-dimethylamino)ethyl methacrylate (DMAEM) and 4VP side chains were formed through RAFT copolymerization of 4VP and DMAEMA monomer in DMF at 70 °C using AIBN as an initiator, and 2-(dodecylthiocarbonothioylthio)-2-methylpropionic acid as a chain transfer agent (CTA). The P4VP-co-PDMAEMA random copolymers with different 4VP-to-DMAEMA molar ratios (see Table 1) were characterized using 1H NMR spectroscopy (Figure 1), FTIR (Figure S1), and GPC (Figure S2). Figure 1 shows the 1H NMR spectrum of the P4VP-co-PDMAEMA (P4) copolymer. The signals at δ 8.5 (peak “1”) and δ 7.0 (peak “2”) originate from the pyridyl group of the 4VP-repeating unit. The peaks at δ 2.89 (peak “5”) are attributed to the 6 protons of the –N(CH_3_)_2_ terminal group of DMAEMA. Compared with the 1H NMR spectrum of P4VPPC-co-PDMAPC (Figure 2), a resonance signal for the 6 protons of the –N(CH_3_)_2_ initiating group is present at 3.3 ppm and 3.8 ppm, demonstrating that a CBMA fragment has been successfully introduced into P4VP-co-PDMAEMA during the β-propiolactone functionalization step. FTIR spectra of the P4VPPC-co-PDMAPC copolymers (Figure 3) are compared to those of pristine P4VP-co-PDMAEMA (Figure S2), which indicates that all of the polymers contain carboxybetaine methacrylate (CBMA) side chains (characteristic peaks: 1724 cm^−1^: C=O stretching; 1594 cm^−1^: COO- asymmetric stretching and 1388 cm^−1^: and COO- symmetric stretching) [10,27]. According to the aforementioned results, we can conclude that well-defined P4VPPC-co-PDMAPC copolymers with different contents of CBMA were successfully synthesized, and furthermore, the surface behaviors and antifouling properties of the asymmetric polymers could be distinctly tuned by the CBMA repeated unit.

### 3.2. Surface Characterization of Polymer Films

**Figure 4:**
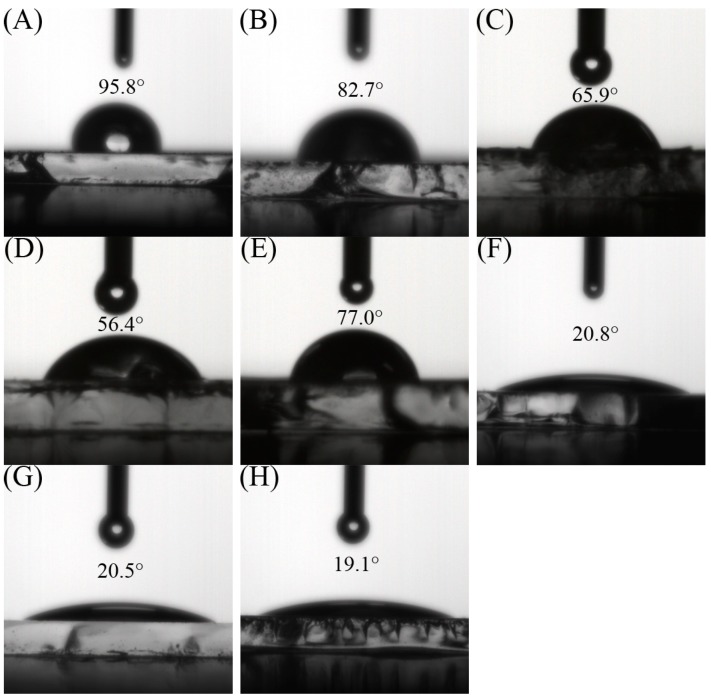
Contact angle images for different polymer films: P4VP (**A**), P1 (**B**), P2 (**C**), P3 (**D**), P4 (**E**), CP20 (**F**), CP40 (**G**), and CP60 (**H**).

The surface property of the P4VP-co-PDMAEMA and P4VPPC-co-PDMAPC films prepared by spin-casting on ITO glasses were evaluated using water contact angle measurements (see Figure 4). The water contact angle for P1, P2, P3, and P4 was 82.7°, 65.9°, 56.4°, and 77.0°, which were much smaller than that of P4VP on the ITO surface (95.8°) [32,33]. The contact angle of P4VP-co-PDMAEMA decreased but the effect is not obvious. After reaction with β-propiolactone, the contact angles for CP20, CP40, and CP60 were decreased to 20.8°, 20.5°, and 19.1°, respectively. It seems that the films from P4VPPC-co-PDMAPC were more hydrophilic than those from P4VP-co-PDMAEMA. Meanwhile, CP60 had a lower contact angle than that of CP40, which could be attributed to the subsequent higher degree of quaternization. In order to increase the surface hydrophilicity, we need to finely control the content of 4VP and DMAEMA side chains, which would affect the quaternary ammonium salting reaction with β-propiolactone.

XPS analysis of the polymer surfaces revealed elemental signals for C, O, N, and Si for all of the ITO adsorbed P4VP-co-PDMAEMA and P4VPPC-co-PDMAPC polymers (see Table 2) [10,34]. As shown in Table 2, an increase in N 1s and a decrease Si 2p signals were observed after polymer adsorption, which indicates that the polymers were adsorbed onto the ITO surface. The surface C/O ratio was greater for samples containing adsorbed polymers, whose values could be used to further demonstrate the presence of polymers on the surfaces. Increased CBMA branching in P4VPPC-co-PDMAPC surfaces resulted in the molar ratio of C/O changing from 4.23 to 3.42 and the N 1s decreasing from 5.61 to 4.41. CP20 gave higher surface C/O ratios than CP60, thus confirming that adsorption efficiency was dependent on the length of the CBMA side chains. The above-mentioned results are consistent with the 1H NMR spectrum and water contact angle on the surfaces.

**Figure 5:**
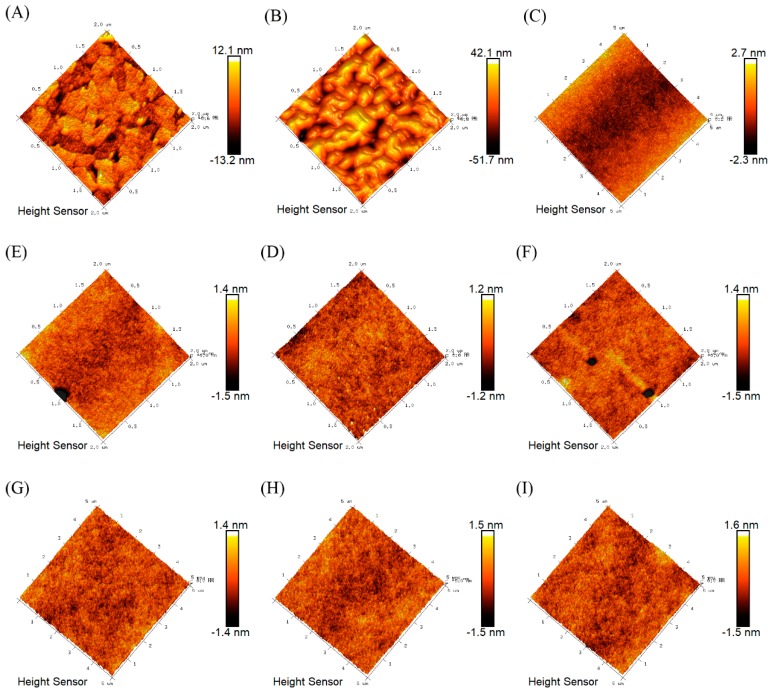
3D AFM images of the surface morphologies of: ITO (**A**), P4VP (**B**), P1 (**C**), P2 (**D**), P3 (**E**), P4 (**F**), CP20 (**G**), CP40 (**H**) and CP60 (**I**).

The morphologies of the bare ITO glass and the polymer surfaces were determined using tapping mode atomic force microscopy to provide information on the surface ordering of the P4VP-co-PDMAEMA and P4VPPC-co-PDMAPC films (see Figure 5) [30]. The root-mean-square (rms) roughness value of the bare ITO surface was 3.73 ± 0.22 nm, and decreased when the ITO surface was coated with polymers. The roughness value of P4VP was 12.23 ± 1.42 nm. Increasing DMAEMA amount of P4VP-co-PDMAEMA resulted in a decrease in roughness (P1 0.79 ± 0.08 nm, P2 0.70 ± 0.11 nm, P3 0.68 ± 0.12 nm, and P4 1.37 ± 0.87 nm), with similar results observed for P4VPPC-co-PDMAPC, the roughness values of CP20, CP40, and CP60 were 0.43 ± 0.09 nm, 0.41 ± 0.11 nm, and 0.44 ± 0.08 nm. These observations are consistent with previous results that high levels of CBMA produced films with more flexible hydrophilic side-chains that could decrease the roughness of the film surface [35,36].

### 3.3. Antifouling Properties of Polymer Thin Films

**Figure 6:**
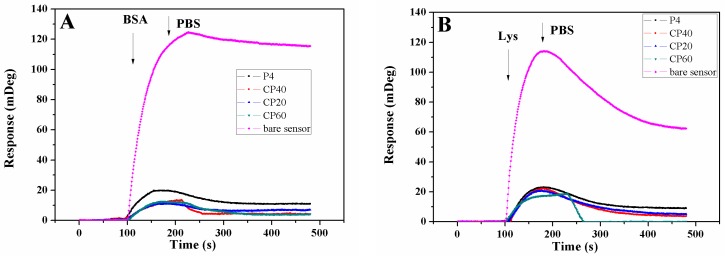
Adsorption profiles of BSA (**A**) and Lys (**B**) on bare sensor for P4, CP20, CP40, and CP60 polymers.

The antifouling properties of the P4VP-co-PDMAEMA and P4VPPC-co-PDMAPC films surfaces were tested using protein adsorption and cell attachment assays. Different proteins exhibited different adsorption patterns for the same surface, with their isoelectric point (PI) playing an important role in their deposition processes. The protein resistance properties of the polymer brush P4VP-co-PDMAEMA and P4VPPC-co-PDMAPC films were investigated using fluorescently labeled BSA and Lys (PI for BSA = 4.8; PI for Lys = 10.7) [12,37]. These results showed that BSA was adsorbed onto the bare ITO and polymer films (Figure 6), thus demonstrating a strong interaction between this protein and the surfaces of these polymer films. However, the P4VPPC-co-PDMAPC films were found to be effective in reducing BSA and Lys absorption. The antifouling effects toward BSA and Lys were found to increase as the amount of CBMA side chain increased. When the zwitterion content was increased to 60% for the CP60 polymer, the BSA adsorption decreased to 3.6% of the levels measured for bare Au, with a similar trend observed where the adsorption of Lys decreased to 0. This result illustrates that the CBMA side chain alone possesses good resistance against proteins. All of the P4VPPC-co-PDMAPC zwitterionic films exhibited almost no adsorption of Lys, with the protein binding levels significantly less than for only P4VP-co-PDMAEMA films. Therefore, the observed reduction in adsorption levels is due to the presence of the zwitterion regions in the films.

**Figure 7:**
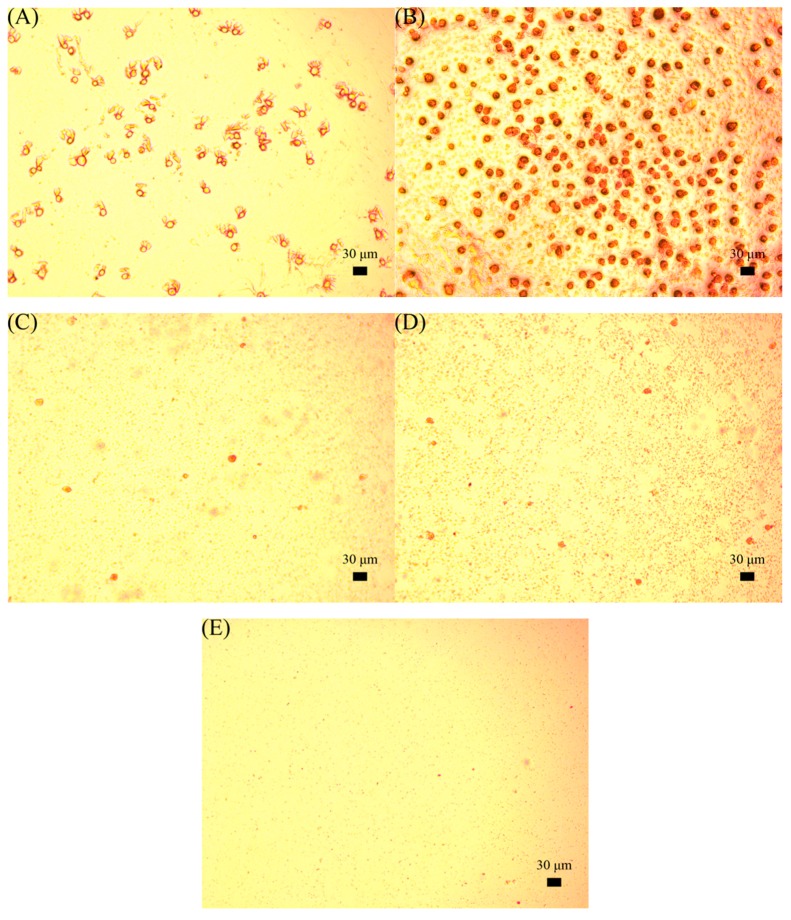
Images of HEK-293T cells grown on ITO (**A**), P4 (**B**), CP20 (**C**), CP40 (**D**), and CP60 (**E**) polymer surfaces, scale bar = 30 μm.

**Figure 8:**
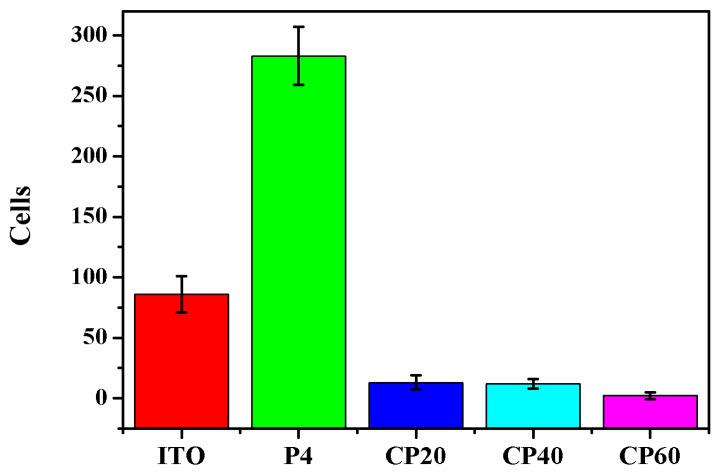
Quantification of cells appearing on different sample surfaces, data are shown as mean ± standard error for three samples.

Based on these results, the antifouling property of the polymer-based films was investigated by evaluating the adhesion of HEK-293T cells via microscopy (Figure 7). The P4VP-co-PDMAEMA polymer films of P4 showed a 50% increase in adsorbed cells in comparison with bare ITO surfaces. The zwitterionic P4VPPC-co-PDMAPC films reduced 90% of HEK-293T cells adhesion. Meanwhile, it was reported that PCB-coated surfaces could reduce the P. aeruginosa and P. putida biofilm formation by 95% in comparison to the bare glass for ATRP biofilms [10]. More impressively in this study, the P4VPPC-co-PDMAPC-coated ITO surface CP60 was shown to reduce cell adsorption by 99%. Figure 8 shows the number of single and clustered cells, quantified to assess the prevalence of nonspecific protein adsorption on the surfaces. The P4VP-co-PDMAEMA polymer surfaces of P4 (283 ± 24) showed a more than three times increase in the increment of adsorbed cells in comparison with ITO surface (86 ± 15). The zwitterionic P4VPPC-co-PDMAPC coating (CP60) reduced 99% of HEK-293T cells adhesion. These results indicate that high antifouling property was established on the basis of control over the polymer architecture, and the addition of 4VP side chains could effectively solve the problem of the hydrolysis of zwitterionic polymers. Through the comparison of their antifouling properties, the polymer containing both 4VP and zwitterionic side chains (CP60) exhibited a pronounced antifouling property against human kidney cell absorption.

## 4. Conclusions

A new strategy has been developed to optimize the protein resistance properties of P4VP modified zwitterionic polymers on ITO surfaces that were prepared using the RAFT copolymerization and quaternary ammonium salting reaction with β-propiolactone processes. The water contact angle and AFM measurements have revealed the smoothness and high hydrophilicity of P4VPPC-co-PDMAPC films, with their protein and cell resistance properties found to be dependent on the ratio of their 4VP and zwitterionic side chains. The P4VPPC-co-PDMAPC copolymer surfaces were shown to display high nonfouling activity against BSA and Lys proteins and human kidney cells, when compared to P4VP or P4VP-co-PDMAEMA polymer surfaces. This study describes a simple but effective approach for preparing nonfouling surfaces, with the antifouling properties of their zwitterion components suggesting that these materials are likely to be useful for the preparation of biomaterials for biosensing and diagnostic applications.

## Figures and Tables

**Table 1:** Structural parameters of the P4VPx-co-PDMAEMA polymer.

Entry ^a^	[M]:[AIBN]	Mn ^b^	Mw/Mn ^b^
P4VP	60:1	2540	1.11
P1	60:1	3870	1.31
P2	180:1	4400	1.15
P3	300:1	6600	1.24
P4	540:1	11000	1.36

^a^ Polymerization temperature: 70 °C. [4VP]:[DMAEMA] = 1:1. ^b^ Measured using GPC analysis at 25 °C in THF.

**Table 2:** XPS-analysis of surface atomic concentrations and molar ratios of bare and polymer-modified ITO surfaces.

Sample	Si 2p (%)	N 1s (%)	C:O
ITO	11.57	0.86	2.36
P4VP	4.97	6.97	8.72
P1	4.85	5.93	4.83
P2	3.76	6.22	5.53
P3	5.10	5.89	4.77
P4	4.02	6.13	4.59
CP20	5.79	5.61	4.23
CP40	5.47	4.57	3.94
CP60	0	4.41	3.42

## Supplementary Materials

The following are available online at https://www.mdpi.com/2079-4991/9/5/706/s1, Figure S1. FTIR spectra of P4VP, P1, P2, P3 and P4, Figure S2. GPC traces of P4VP, P1, P2, P3 and P4 in THF, Figure S3. Survey XPS data for Bare ITO surface, Figure S4. Survey XPS data for P4VP polymer surface, Figure S5. Survey XPS data for P1 polymer surface, Figure S6. Survey XPS data for P2 polymer brush surface, Figure S7. Survey XPS data for P3 polymer surface, Figure S8. Survey XPS data for P4 polymer surface, Figure S9. Survey XPS data for CP20 polymer surface, Figure S10. Survey XPS data for CP40 polymer surface, Figure S11. Survey XPS data for CP60 polymer surface.
