# Supplementary material for: P4VP Modified Zwitterionic Polymer for the Preparation of Antifouling Functionalized Surfaces
Source: Nanomaterials (Basel). 2019 May 7;9(5):706. doi: 10.3390/nano9050706 (PMC6566957; doi:10.3390/nano9050706)
Supplement: Supplementary file 1 [file nanomaterials-09-00706-s001.pdf]

Supplementary Material
P4VP modified Zwitterionic Polymer for the Preparation of Antifouling Functionalized Surfaces

Chaoqun Wu [1], Yudan Zhou [1], Haitao Wang [1] and Jianhua Hu [1,*]

[1] State Key Laboratory of Molecular Engineering of Polymers, Department of Macromolecular Science, Fudan University, 2005 Songhu Road, Shanghai, 200438, People's Republic of China

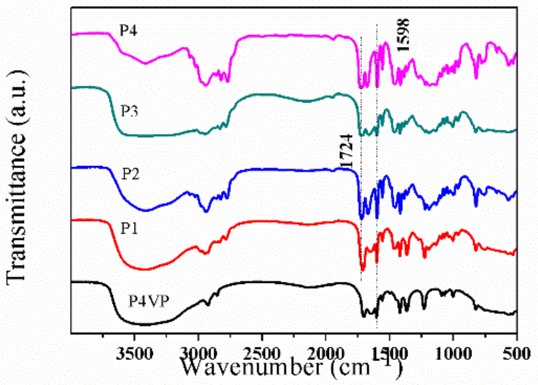

Figure S1. FTIR spectra of P4VP, P1, P2, P3 and P4.

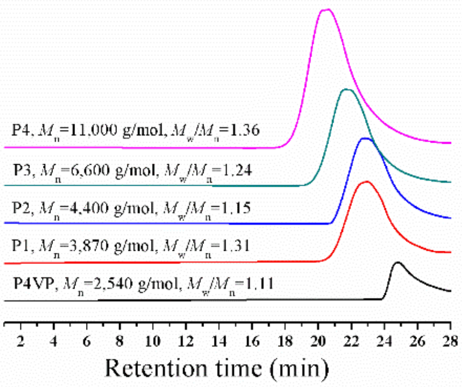

Figure S2. GPC traces of P4VP, P1, P2, P3 and P4 in THF.

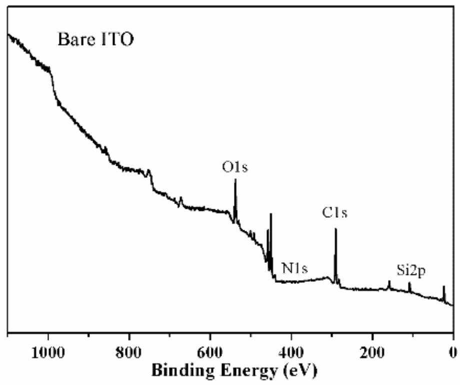

Figure S3. Survey XPS data for Bare ITO surface.

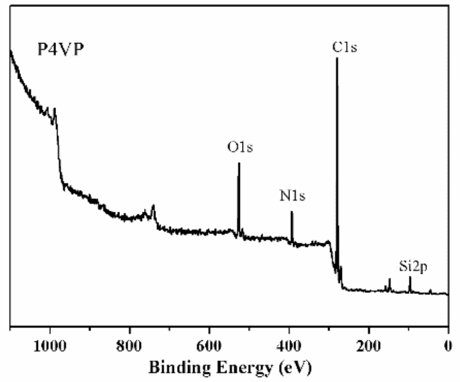

Figure S4. Survey XPS data for P4VP polymer surface.

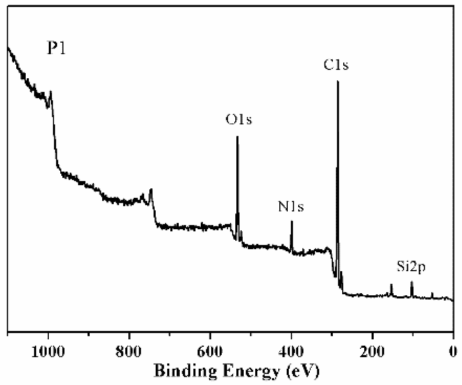

Figure S5. Survey XPS data for P1 polymer surface.

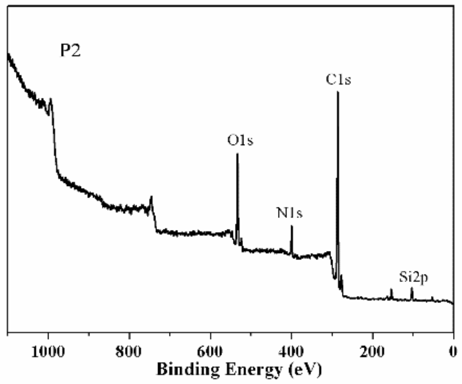

Figure S6. Survey XPS data for P2 polymer brush surface.

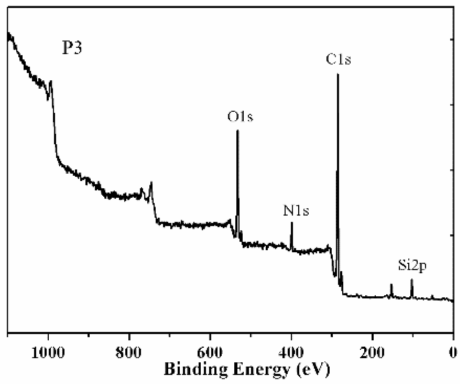

Figure S7. Survey XPS data for P3 polymer surface.

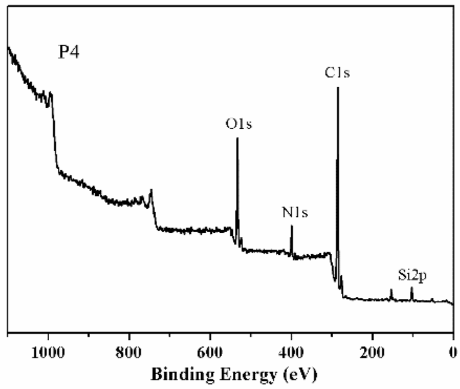

Figure S8. Survey XPS data for P4 polymer surface.

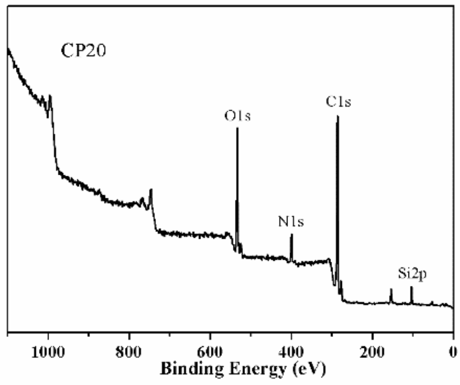

Figure S9. Survey XPS data for CP20 polymer surface.

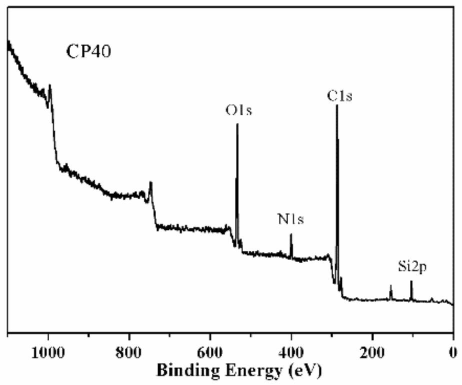

Figure S10. Survey XPS data for CP40 polymer surface.

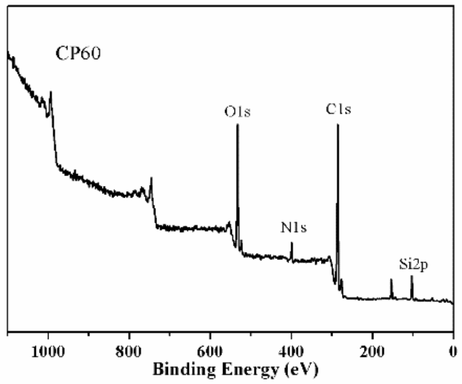

Figure S11. Survey XPS data for CP60 polymer surface.
